# Dual‐frequency ultrasound for ultrasonic‐assisted esterification

**DOI:** 10.1002/fsn3.1115

**Published:** 2019-06-25

**Authors:** Elahe Abedi, Kiana Pourmohammadi, Sahar Abbasi

**Affiliations:** ^1^ Department of Food Science and Technology, College of Agriculture Fasa University Fasa Iran; ^2^ Department of Food Science and Technology Sarvestan Azad University Sarvestan Iran

**Keywords:** Acetylation, dual‐frequency ultrasonic, optimization, wheat starch

## Abstract

The optimization of wheat starch esterification (acetylation) with a high degree of substitution was performed through response surface methodology (RSM) via various concentrations of reagents (acetic anhydride), pHs, and temperatures under various ultrasonication frequencies (25, 40, and 25 + 40 kHz). According to RSM methodology, optimized samples were selected by achieving high degrees of substitution at various frequencies, temperatures, and pHs. Solubility, swelling, X‐ray, RVA, DSC, freeze–thaw stability, texture, and SEM analysis of the optimized samples were performed at three frequencies. X‐ray pattern exhibited a more significant reduction in the crystallinity percentage of esterified starch at frequency 25 + 40 kHz compared with 25 kHz, 40 kHz, and native starch. According to DSC analysis, To, Tp, Tc, and enthalpy of gelatinization (ΔH gel) were lower in AC at frequency 25 + 40 kHz compared with AC at frequency 25 and 40 kHz and N starches. According to morphology analysis, in acetylated starches at 25 and 40 kHz, the surfaces and small granules underwent more damage, whereas in 25 + 40 kHz, large granules were more affected than small granules. Upon acetylation, freeze–thaw stability and textural properties of the starch significantly increased and decreased, respectively. The peak and final viscosity of acetylated starch increased (25 + 40 kHz ˃ 25 kHz ˃ 40 kHz ˃ N starch).

## 1 INTRODUCTION

Starch is an accessible, thickening, and texturizing stabilizer, and of paramount importance when it comes to augmenting the overall quality of the products, reducing costs, and facilitating the processing procedure (Berski et al., 2011; Choi & Kerr, 2003); however, due to the high affinity toward retrogradation, its application is restricted in certain food products (Kaur, Ariffin, Bhat, & Karim, 2012; Kaur, Sandhu, & Lim, 2010). In frozen foods and desserts containing starch, resistance to syneresis of water, resistance to freeze and thaw, and reduction in retrogradation are important issues. In this regard, native starch was modified to overcome this limitation. By introducing the functional groups into the starch molecules, chemically modified starches, such as oxidized, hydroxypropylated, and acetylated starches, were employed. When starch is modified, its gelatinization, pasting, and retrogradation characteristics undergo different changes (Kaur et al., 2012, 2010; Singh, Kaur, & McCarthy, 2007). Acetylated starch (AC) was introduced with CH_3_CO group which caused a cleavage in the hydrogen bonds inside the starch chain and resulted in amphiphilic properties (Hong, Chen, Zeng, & Han, 2016). AC with a low degree of substitution (DS) is used as an emulsifier, coating, and thickening agent, which is stable and resistant to retrogradation (Chi et al., 2008; Singh, Chawla, & Singh, 2004). Moreover, in such kinds of modified starch, solubility, swelling power, viscosity, hardness, adhesiveness, cohesiveness, and translucency of the gels are increased, while the initial gelatinization temperature is reduced (González & Perez, 2002). The replacement of the modifier groups may boost the free movement of the starch chains within the amorphous regions in the granule, owing to the disruptions taking place among the inter‐ and intramolecular hydrogen bonds. The weakened internal bond structure in the starch granules, due to the derivatized modifier groups, enhances the freeze–thaw stability, reduces the gelatinization temperature, generates high levels of peak viscosity, and leads to starch paste clarity (Han, 2010; Han, Lee, Lim, & Lim, 2005).

Ultrasonic treatments can accelerate chemical reaction and increase yields. Under sonication, the surface area of starch granules increases due to the formation of channels and/or holes on the surface and inside the granules (Huang, Li, & Fu, 2007). Several modifications have been performed upon ultrasonication, including acetylation of dioscorea starch following ultrasound treatment (Zhang, Zuo, Wu, Wang, & Gao, 2012), octenyl succinate starch (Chen, Huang, Fu, & Luo, 2014), carboxymethyl starch (Gao et al., 2011; Shi & Hu, 2013), and octenylsuccinates of carboxymethyl starch (Čížová, Sroková, Sasinková, Malovíková, & Ebringerová, 2008). However, there is no information about the acetylation of starch under various frequencies of ultrasonication. The aim of this study was to optimize the AC wheat starch in various parameters such as acetic anhydride concentration, pH, and temperature under frequencies (25, 40, and 25 + 40 kHz) of bath ultrasound; the optimization was further investigated on the amount of modification (molar substitution) in the modified starch. Based on the minimal reagent consumption, the optimized samples were selected and their physicochemical properties (solubility, water absorption), X‐ray diffraction, scanning electron microscopy (SEM), Rapid Visco Analyzer (RVA), differential scanning calorimetry (DSC), and Texture Profile Analyzer (TPA) were investigated at three frequencies (25, 40, and 25 + 40 kHz). The freeze and thaw stability of AC wheat starch was further compared with that made with native wheat starch.

## 2 MATERIALS AND METHODS

### 2.1 Materials

Native wheat starch was obtained from Fars Glucosin Company, Shiraz, Iran. The moisture, fat, ash, and protein of native (N) and AC wheat starch prepared under ultrasonication were specified along with the standard methods of AACC, 2000. Amylose content (%) was calculated by iodine method (Pourmohammadi, Abedi, Hashemi, & Torri, 2018).

### 2.2 Acetylation of wheat starch

We added 100 g of starch to 224 ml of distilled water and stirred for 1 hr at 24°C. The pH was set to 6.5, 8, and 9.5 with NaOH solution (1 M), and acetic anhydride (4, 6 and 8%) was slowly added to the suspension at various pHs. Whole acetylation reaction was performed at 10 min. The suspensions were sonicated at an amplitude of 24% (with an input power of up to 400 W and working frequencies of 25, 40, and 25 + 40 kHz) at 45, 60, and 75°C. The time duration of sonication in each frequency was 5 min (25 kHz: 5 min, 40 kHz: 5 min, and 25 + 40 kHz: 5 min). pH was then maintained at 4.5 with HCl solution (1 M) for 5 min. All the experiments were performed in bakers placed at known positions in ultrasonic bath (Pacisa SA) equipped with a temperature controlling system. The ultrasonic bath with internal dimensions of 300 × 150 × 150 mm^3 ^consisted of a rectangular tank containing four transducers at the bottom. The suspensions were completely washed, lyophilized, and milled using a laboratory mill (AlexanderWerk, Model WEL82), and manually sieved to obtain particle sizes < 40 μm (Wojeicchowski, Siqueira, Lacerda, Schnitzler, & Demiate, 2018).

### 2.3 Acetyl percentage

The titrimetric method of Mbougueng, Tenin, Scher, and Tchiégang (2012) was employed in order to specify the acetyl group percentage (AC%) and (DS). AC (5.0 g), 50 ml of distilled water, and 24 ml of 0.45 M NaOH were added in a 240‐mL flask and mixed for 30 min at room temperature. The surplus alkali was back‐titrated using 0.2 M HCl and phenolphthalein as an indicator. The reaction mixture was put to stand for 2 hr, where the alkali was removed from the titrated sample. The native starch was also used as a blank sample. Initially, the acetyl (%) and DS were calculated according to Equations (1) and (2), respectively:

(1) $$\%\;\text{Acetylation}=[\left(V_{\text{B}}-V_{\text{S}}\right)\times N_{\text{HCl}}\times 0.043\times 100]/W$$

*V*_B_ and *V*_S_ are the volumes of the control and modified samples (0.2 M HCl basis), respectively.

*N*_HCl _is the normality of the consumed acid.

*W* is the weight of the sample.

(2) DS=(162×Acetyl%)/[4300-(42×Acetyl%)]

### 2.4 X‐ray diffraction

To obtain a relative humidity of 75%, all starch samples were primarily placed, for 5 days, in a relative humidity box containing supersaturated NaCl at room temperature. X‐ray diffraction pattern of the samples was determined by use of an X‐ray diffractometer (Model D8 Advance, Germany) (Li et al., 2014). Utilizing the instrument software (EVA, Version 9.0), the degree of starch crystallinity was specified through dividing the area under the peaks by the total curve area.

### 2.5 Solubility and water absorption

The method of Li et al. (2014) was used to determine water swelling and solubility of the samples with a slight modification. Native and modified wheat starches (1 g) and distilled water (30 ml) were added into a centrifuge tube, and, using a vortex mixer set at high speed, they were shaken at room temperature for 5 min. Each tube was incubated in water bath for 30 min at 95°C followed by centrifugation for 15 min at 3000 g. The weights of the pellet and dried supernatants (at 120°C for about 2 hr) were further obtained. The water solubility and swelling values of the native and modified samples were calculated using Equations (3) and (4), respectively.

(3) $$\text{Water solubility}\left(\%\right)=\text{weight of dissolved solids in supernatant/weight of dry sample solids in the original sample}\times 100$$

(4) $$\text{Water swelling}\;\left(\%\right)=\left(\text{weight of sediment}\times 100\right)/\left(\;\text{weight of dry sample solids}\times \left(100-\text{solubility}\right)\right)$$

### 2.6 Pasting properties

Using the method described by Pourmohammadi et al. (2018), the pasting properties of the native and modified wheat starches were determined through RVA (Newport Scientific Pty. Ltd) interfaced with a personal computer.

### 2.7 Thermal properties

The gelatinization properties of the native and modified wheat starches were analyzed using a DSC instrument (OIT‐5000; Sanaf) equipped with STAR software making using of Pourmohammadi et al. (2018) method. The device was calibrated with indium and mercury, and an empty pan was used as a reference. Native and modified wheat starches (10 g) were weighed into the standard aluminum pan. Distilled water (30 ml) was added, and the pans were sealed and equilibrated for 24 hr at room temperature. After that, the starch slurry was gelatinized in the DSC instrument, by heating at 6°C/min, from 10 to 160°C. Following heat treatment, the samples were cooled to 25°C and then removed from the DSC. The changes in enthalpy (ΔH in J/g of dry starch), onset temperature (*T*_o_), peak temperature (*T*_p_), and conclusion temperature (*T*_c_) for gelatinization were obtained from the exotherm DSC curves.

### 2.8 Microstructure determination

The microstructure of the modified starch was specified using SEM (Model Leica Cambridge) by use of the method proposed by Pourmohammadi et al. (2018).

### 2.9 Textural analysis

Textural properties of starch gels were specified using a Texture Profile Analyzer (TA Plus; Stable Micro System). To prepare the paste of gelatinized starch, 10 g of starch was added to 100 ml of distilled water, where the slurry remained in water bath (95°C for 30 min). The hot paste was collected and transferred into a cylindrical plastic container of 10 mm diameter and 10 mm height, and kept at 4°C for 24 hr. The gels were then removed from the container and tested for their textural properties. Utilizing a cylindrical aluminum plunger with a diameter of 10 mm, the gels were packed at a test speed of 2 mm/s, pretest and post‐test speed of 5 mm/s, time interval of 10 s, and strain deformation of 24%. The recorded force–time plots were analyzed for the following: hardness (N), springiness (Length 2/ Length 1), cohesiveness, the ratio of the areas of the two resistance peaks (A2/A1), and gumminess according to Abedi, Majzoobi, Farahnaky, Pourmohammadi, and Mahmoudi (2018). Data were analyzed three times, and the results were averaged.

### 2.10 Freeze–thaw stability

With slight modifications in the methods described by Lawal (2010), we determined the syneresis of the starch gels belonging to the native and modified starches during cold and frozen storage. A starch suspension (5% dry basis, w/w) was heated at 95°C under constant agitation for 1 hr. 20 g of noodle strands belonging to N and AC starch was centrifuged at 2000 g for 10 min to remove free water. The free water (supernatant) was decanted, and the tubes containing starch paste were exposed to freeze and thaw cycles, followed by centrifugation at 3,500 g for 30 min. The freezing process was done at −18°C for 24 hr, and the melting process was carried out at 30°C for 4 hr. The two processes were repeated for 8 cycles, in each of which the isolated water was determined. The weight of water was taken, and the amount of syneresis was calculated as the percentage of water separated as Equation (5):

(5) $$\text{Syneresis}\left(\%\right)=\text{Water separated}\left(g\right)\times 100)/\text{Total weight of sample}\left(g\right)$$

### 2.11 Statistical analysis

So as to specify the regression coefficients, the Design‐Expert (version 6.0.5) methodology (Abedi, Sahari, Barzegar, & Azizi, 2015) and statistical software packages SAS 9.1 (SAS Institute) were made. A Box–Behnken design from the response surface was used to study the effect of three different factors on the acetylation degrees of wheat starch. Factors associated with acetylation were acetic anhydride (4, 6, and 8%), pH (6.5, 8, and 9.5), and temperature (45, 60, and 75°C). The experimental data were fitted in accordance with Equation (6) as a second‐order polynomial equation, including the linear and interaction effects of each factor:

(6) $$Y=\beta_{0}+\sum_{i=1}^{k}\beta_{i}X_{i}+\sum_{i=1}^{k}\beta_{\mathrm{ii}}X_{i}^{2}+\sum_{\begin{array}{c} i=1\\ i<j \end{array}}^{k-1}\sum_{j=2}^{k}\beta_{\mathrm{ij}}X_{i}X_{j}$$

where *Y* is the predicted response, *X_i_* and *X_j_* are independent factors, b0 is the offset term, bi is the ith linear coefficient, bii is the ith quadratic coefficient, and bij is the ijth interaction coefficient. All analyses were obtained in triplicates and reported as mean values.

## 3 RESULTS AND DISCUSSION

### 3.1 Molar substitution of AC wheat starch

**Figure 1:**
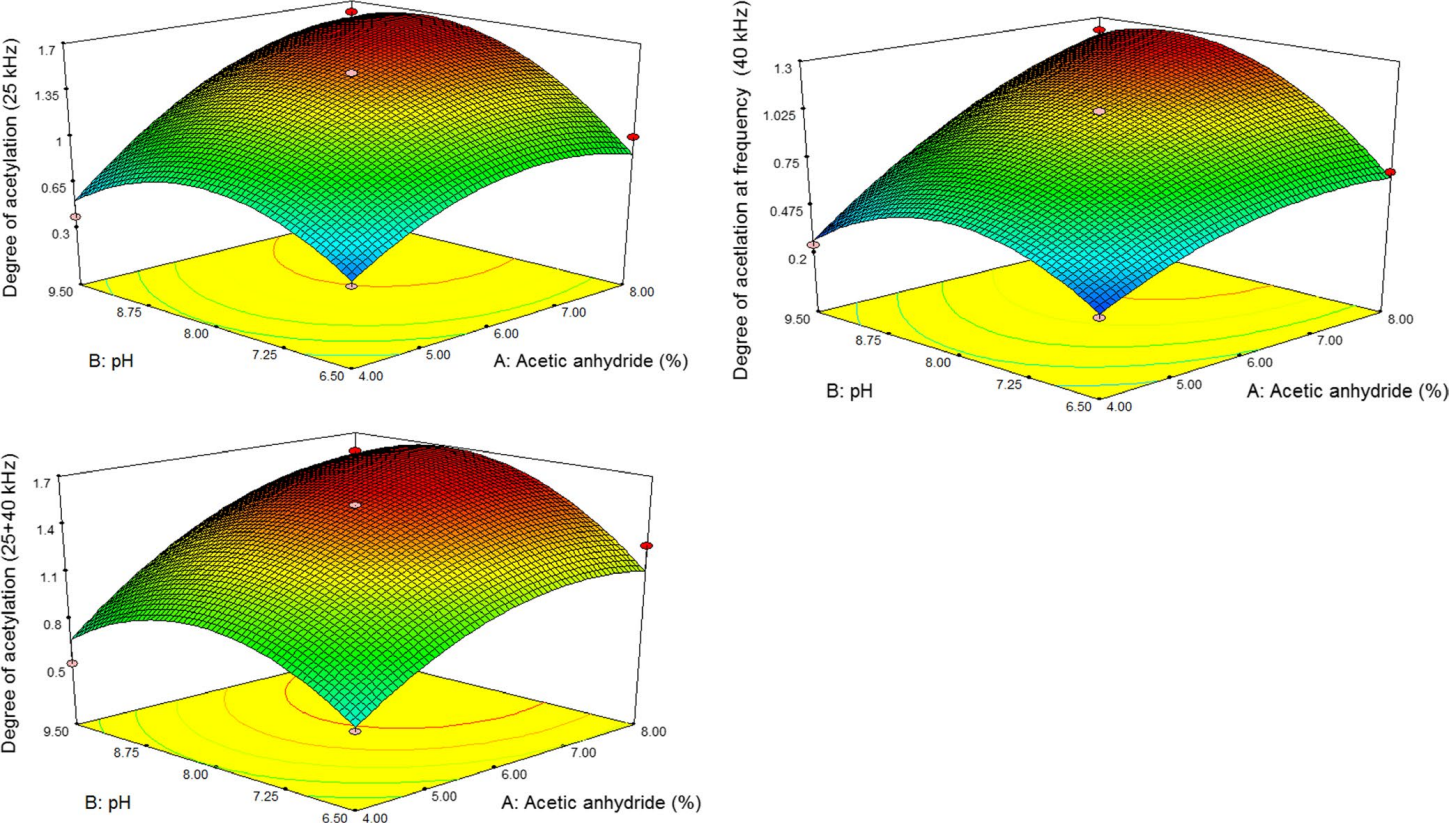
The effect of acetic anhydride (4, 6, and 8%), pH (6.5, 8, and 9.5), and temperature (45, 60, and 75°C) on the degree of wheat acetylation (A) at frequencies 25 kHz, 40 kHz, and 25 + 40 kHz. Lack of fit: not significant

Concerning the AC wheat starch at sonication frequencies of 25, 40, and 25 + 40 kHz, optimization was done in order to investigate the effect of acetic anhydride content, pH, temperature, and ultrasonic frequency on the amount of modification or molar substitution of the modified starch (Figure 1).

(7) $$\text{Degree of acetylation}\left(\%\right)\text{at frequency 25 kHz}=+1.48+0.37\times A+0.18\times B+0.017\times C+0.17\times A\times B+0.16\times A\times C+0.043\times B\times C-0.26\times A^{2}-0.37\times B2-0.50\times C^{2}$$

(8) $$\text{Degree of acetylation}\left(\%\right)\text{at frequency 40 kHz}=+1.02+0.32\times A+0.13\times B+0.13\times C+0.15\times A\times B+0.15\times A\times C+0.12\times B\times C-0.12\times A^{2}-0.29\times B^{2}-0.36\times C^{2}$$

(9) $$\text{Degree of acetylation}\left(\%\right)\text{at frequency}\;25+40\;\text{kHz}=+1.52+0.35\times A+0.12\times B-0.14\times C+0.093\times A\times B+0.080\times A\times C+0.001\times B\times C-0.21\times A^{2}-0.33\times B^{2}-0.63\times C^{2}$$

Optimized samples were selected based on minimum reagent consumption (acetic anhydride with achieving to maximum modification). According to response surface methodology (RSM) analysis, the optimized AC wheat starch was acetic anhydride (5%), pH 8.5, and temperature 60°C with 1.22%, 1.37%, and 1.02% acetylation for 25 kHz, 25 + 40 kHz, and 40 kHz, respectively.

As a result of Equations ((6), (7), (8)), the effect of A (acetic anhydride content) > B (pH) > C (reaction temperature) is on route to the maximum acetylation. The negative effect of temperature at dual frequency (25 + 40 kHz) was more than 25 and 40 kHz; however, the effect of temperature at 25 kHz frequency was the same as 40 kHz. The addition–elimination is the main mechanism for acetylation reaction, in which hydroxyl groups are converted to acetyl. The hydroxyl groups presented different levels of reactivity. The most reactive group was the hydroxyl of Carbon 6, followed by those of Carbons 2 and 3 (Wojeicchowski et al., 2018). The C* = *O bond of the acetyl group was substituted in all amorphous areas and at the outer lamellae of crystalline sections due to the poor ability of acetic anhydride to penetrate granule structures (Singh et al., 2007).

The differences in the DS of modified starch are related to several parameters, such as starch source, amylose‐to‐amylopectin ratio, degree of crystallinity, molecular weight (MW) of amylose and amylopectin, granular size, presence of lipids, and reaction medium factors, such as pH, temperature, reaction time, mixing uniformity, and reagent content during the modification of the starches (Ayucitra, 2012; González & Perez, 2002; Halal et al., 2015; Salcedo Mendoza, Hernández RuyDíaz, & Fernández Quintero, 2016; Singh et al., 2007; Sodhi & Singh, 2005; Wang & Wang, 2002).

### 3.2 Effect of ultrasonication

Under sonification, DS was increased since the solubility and swelling of granules were improved as a result of sufficient contact with reagents, which enhanced the homogeneity of the reactants. Collapse of cavitation bubbles upon ultrasonic treatment is capable of mechanically damaging and rupturing the starch granules and breaking the chains of polymers due to high‐pressure gradients, high local velocities of liquid layers, shearing force, and microstreaming. According to Amini, Razavi, and Mortazavi (2015) and Zhu (2015), ultrasound creating fracture, pore, and crack on glanular surfaces may enhance the surface area of starch, facilitate the penetration of esterifying agents (acetyl) into the granular structure, and accelerate the chemical reactions. The frequency of ultrasound is a key factor for enhancing cavitational bubble due to certain reasons (Abedi, Sahari, & Hashemi, 2017): (a) Cavitation yield decreases with the increase in frequency, which is attributed to the scattering, attenuation, and shortening of the acoustic cycle at high frequencies (Abedi et al., 2017). (b) Instable cavitation at low frequencies causes the bubble to collapse very quickly and violently, resulting in more rapid agitation and mass transfer (Abedi et al., 2017).

### 3.3 Effect of temperature

Temperature had a progressive effect on the DS of the modified starch, which is attributed to the swelling of starch granules, diffusion of the esterifying agents, mobility of reactant molecules for reaction with amorphous regions, destruction of the crystalline regions of the starch granules, and conversion to amorphous regions (Han et al., 2012). This phenomenon can simplify the acceptability of granules in acetic anhydride and propylene glycol percolation. Temperature is an important factor, yet a very high temperature is not conducive to the absorption of the esterifying agent (acetyl) by the starch. This is because of the exothermic esterification reaction of starch (Han et al., 2012; Singh et al., 2007) and the facilitation of the gelatinization layer, which inhibits the contact of reagents with starch molecules (Han et al., 2012; Singh et al., 2007).

### 3.4 Effect of pH

DS increased following the addition of NaOH to the reaction medium, when the pH was set to 6.5, 8, and 9.5. Alkaline pH was able to enhance the DS owing to the disruption in the hydrogen bonds between molecules in amorphous and crystalline regions, and the facilitation of the percolation and penetration of the esterifying groups into the starch granular structure (Halal et al., 2015; Han et al., 2012). Although pH plays a major factor in the DS improvement of acetylation, very high pHs can diminish DS due to the hydrolyzation of acetic anhydride and the inhibition effect of gelatinized layer on the efficient contact between starch and acetic anhydride (Halal et al., 2015; Han et al., 2012). Various botanical starches have been acetylated with different degrees of acetylation, such as 0.104%–0.184% for maize starch (Singh et al., 2004), 0.081 for waxy maize starch (Wang & Wang, 2002), 0.087%–0.118% (Sodhi & Singh, 2005) for rice starch, 0.041%, 0.059, and 0.076 for tapioca starch (Babic et al., 2007), 1.85, 0.85, and 2.79% for corn starch (Chi et al., 2008), 0.11 and 0.05 with 6 and 8% acetic acid consumption for oat starch (Mirmoghtadaie, Kadivar, & Shahedi, 2009), and 0.9 and 2.7% for barely starch (Bello‐Pérez, Agama‐Acevedo, Zamudio‐Flores, Mendez‐Montealvo, & Rodriguez‐Ambriz, 2010), 0.08%–0.21% for corn starch (Ayucitra, 2012). Potato, oat, wheat, maize, and rice starches presented significant differences in their DS when acetylated under similar reaction conditions (Mirmoghtadaie et al., 2009; Singh et al., 2007).

The high molar substitution acetylation in this study was interpreted by reaction medium under ultrasonic process, which affects the amylose regions. The extent of modification by ultrasonication in the crystallinity of starch granules depends on experimental conditions and the type of starch (Zhu, 2015), and requires the introduction of acetyl group throughout the amylose or amylopectin sections. According to Singh et al. (2004), with low amylose content (low amylose/amylopectin ratio) a very high DS of acetylation can be observed. Surface of granule is another major factor affecting DS. Furthermore, González and Perez (2002) interpreted that the low degree of substitution in rice starch might be due to the lack of enough large inner channels or granular surface pores which can facilitate the access of acetic anhydride to the interior of the granule.

### 3.5 The crystalline structure

**Figure 2:**
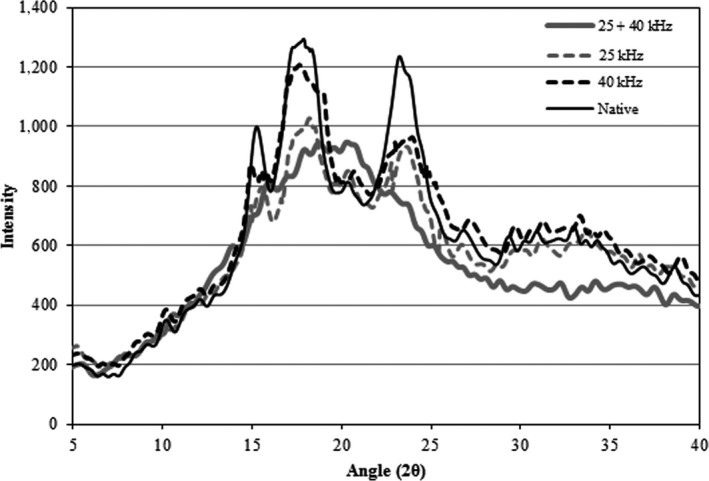
X‐ray diffraction of native and acetylated wheat starch at frequencies 25 kHz, 40 kHz, and 25 + 40 kHz

The X‐ray diffraction pattern of native and modified acetylated wheat starches is presented in Figure 2. X‐ray diffraction analysis estimates the crystalline structure and the amount of crystal existing in the starch granules. The structure is related to amylopectin double helix. The results showed that the X‐ray pattern of wheat starch was the same as other cereals (A pattern). The native starch had sharp diffraction peaks at 15°, 17°, 18°, 23° (2θ), indicating the typical A pattern of cereal starch (Zobel, Young, & Rocca, 1988). Following the esterification process, the degree of crystallinity was significantly reduced (*p* < 0.05) from *N* (36.89 ± 0.62) to AC at 25 kHz (17.80 ± 0.38), 40 kHz (22.43 ± 0.42), and 25 + 40 kHz (13.57 ± 0.41) frequencies of wheat starches. The X‐ray diffraction showed that with acetylation, the structures of native starch were destroyed, and new structures of esterified starches were formed. Crystalline regions are the main factors preventing the loss of granular structure during reaction with reagents, and preserving the integrity of granular structure (Sha et al., 2012; Singh et al., 2007). Moreover, it is to be noted that starch granules in amorphous regions are more susceptible to reagents during the modification process, while crystalline regions remain intact. Acetylation under ultrasonic conditions created fractures and cracks on granular surfaces, thereby facilitating the penetration of acetic anhydride for reaction with amorphous (granule composed of amylose) and crystalline regions. The amylose content of *N* (26.30 ± 0.26) was reduced to 12.68 ± 0.12, 18.93 ± 0.24, and 9.23 ± 0.36 following acetylation at ultrasound frequencies 25, 40, and 25 + 40 kHz, respectively. This means that amorphous regions reacted with acetic anhydride, a finding which is in accordance with Diop, Li, Xie, and Shi (2011); Simsek, Ovando‐Martínez, Whitney, and Bello‐Pérez (2012); Mbougueng et al. (2012); Wani, Sogi, and Gill (2012).

### 3.6 Solubility and water absorption

**Figure 3:**
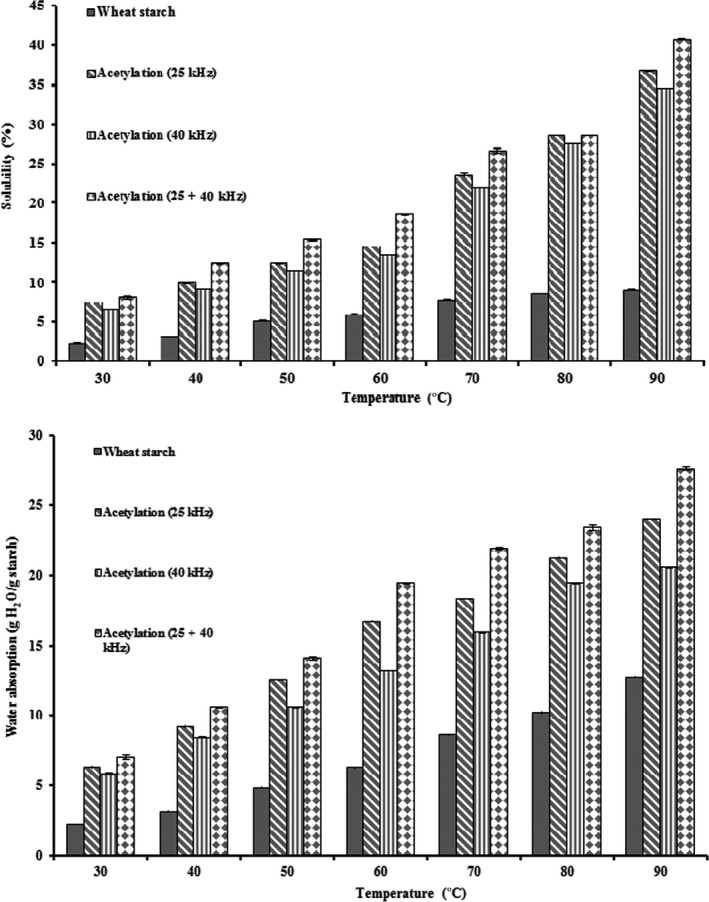
Solubility and water absorption of native and acetylated wheat starch at frequencies 25 kHz, 40 kHz, and 25 + 40 kHz

Figure 3 illustrates the water absorption (WA) and solubility of native and acetylated wheat starch at different temperatures. The solubility and WA of N and AC wheat starch at various frequencies differed significantly (*p* < 0.05) at all measured temperatures. The solubility and water absorption of AC wheat starch at frequencies 25, 40, and 25 + 40 kHz were 7.37–36.77 and 6.30–23.98, 6.57–34.49, and 5.83–20.94, and 8–40.77 and 7.03–27.60, respectively, which is much higher than N wheat starch (2.28–9.03 and 2.28–12.72 at temperatures ranging between 30 and 90°C, respectively). Similar increasing trends in solubility and WA due to acetylation have been reported for potato and corn starches (Singh et al., 2004), waxy and normal maize (Liu, Ramsden, & Corke, 1999), small and large wheat starch (Van Hung & Morita, 2005a), tapioca (Babic et al., 2007), normal and waxy rice starch (Liu et al., 1999), rice starch (González & Perez, 2002; Sodhi & Singh, 2005), barely (Bello‐Pérez et al., 2010; Halal et al., 2015), sweet potato (Lee & Yoo, 2009), oat (Mirmoghtadaie et al., 2009), and corn (Diop et al., 2011; Han et al., 2012). Further reported is the increasing pattern of swelling and solubility properties of various starch sources under ultrasonic processes (Chan, Bhat, & Karim, 2010; Herceg et al., 2010; Luo et al., 2008; Montalbo‐Lomboy et al., 2010; Režek Jambrak et al., 2010; Sujka & Jamroz, 2013; Zheng et al., 2013). Such increase is probably due to the destruction, disorganization, and reduction in the crystallinity degree of starch granules.

The acetyl group disorganized the starch components upon acetylation due to the following: (a) disruption of hydrogen bonds in the starch granules and facilitation of water access to the amorphous region (Babic et al., 2007; González & Perez, 2002; Liu et al., 1999), (b) repulsion force between starch molecules as well as starch chain, (c) partial depolymerization principally in the amylopectin, reducing the average molecular weight (MW) (Bello‐Pérez et al., 2010; González & Perez, 2002), (d) loss of granule crystallinity based on the X‐ray diffraction pattern (Bello‐Pérez et al., 2010; Van Hung & Morita, 2005a, 2005b; Liu et al., 1999), and (e) reduced interactions between the starch chains due to the introduction of acetyl groups, solubilizing and releasing the amylose to the exterior of the swollen starch granule (Salcedo Mendoza et al., 2016). According to Figure 3, temperature had a progressing effect on solubility and swelling of N and AC starch. The increase in the temperature of the medium induces the thermodynamic activity of starch molecules; moreover, a high granular mobility can increase the water penetration in the amorphous regions of starch granules and destroy the crystalline regions as well (Mirmoghtadaie et al., 2009).

### 3.7 The pasting properties

**Figure 4:**
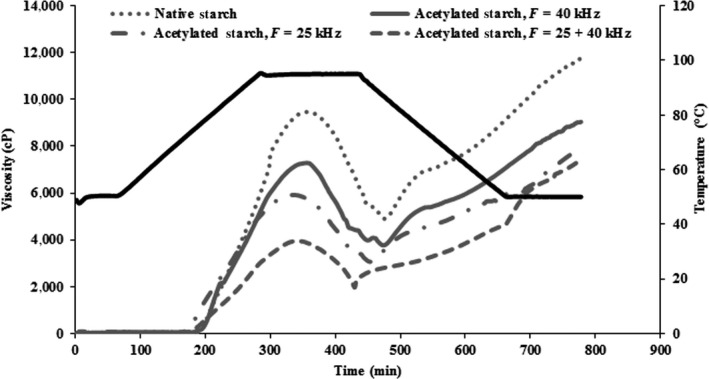
The RVA profiles in native (1) and acetylated wheat starch at frequencies 25 kHz (3), 40 kHz (2), and 25 + 40 kHz (4)

Figure 4 and Table 1 present the RVA profile of N and AC starch suspensions (5%, w/w) at various frequencies (25, 40, and 25 + 40 kHz) during heating from 30 to 90°C. The peak viscosity is indicated by the maximum swelling of the majority of starch granules and their subsequent collapse (Van Hung & Morita, 2005a, b). The peak and final viscosity of native starch (3,757 ± 48 and 7,034 ± 34) were found to be significantly (*p* < 0.05) lower than AC starches at frequencies 25 (7,188 ± 32 and 8,851 ± 31), 40 (5,846 ± 65 and 7,648 ± 66), and 25 + 40 kHz (9,423 ± 112 and 11,174 ± 131). The AC at frequency 25 + 40 kHz (50.9 ± 1.2) had lower pasting temperatures compared with 25 kHz (58.3 ± 1.4), 40 kHz (64.4 ± 1.2), and *N* (71.6 ± 1.6). These findings are in accordance with the results of Li et al. (2014) where the starch with higher amylose content reduced the gelatinization temperatures due to less crystalline and more amorphous regions (Li et al., 2014). These results are in accordance with certain other research (González & Perez, 2002; Van Hung & Morita, 2005a, b; Liu et al., 1999; Salcedo Mendoza et al., 2016). In these studies, the modification of starch by acetylation increased the peak viscosity due to (a) the decrease in the strength of associative intermolecular forces in the amorphous regions of the granules, (b) lower degrees of crystallinity in AC compared with N wheat starches, (c) increase in solubility and swelling power (Figures 3 and 4), (d) the solubilizing amylose released outside the swollen starch granule (Han, 2010; Salcedo Mendoza et al., 2016), and (e) improvement in the hydrophilic capacity of the reorganized structures (Han, 2010; Salcedo Mendoza et al., 2016). On the other hand, compared with the native starches, the AC wheat starches presented better stability during heating. Reassociation of amylose molecules occur when leached out from swollen starch granule, thus providing a higher resistance to thermal or mechanical forces (Colussi et al., 2015).

**Table 1:** Pasting profile (RVA), thermal (DSC), and textural (TPA) analysis of native (N), acetylated (AC), wheat (dry basis) starches

	RVA parameters					Thermal parameters				TPA parameters				
	Pasting temperature (°C)	Peak viscosity (mPa•s)	Final viscosity (mPa•s)	Breakdown viscosity (mPa•s)	Setback viscosity (mPa•s)	*T*_0_ (°C)	*T*_P_ (°C)	*T*_C_ (°C)	ΔH (J/g)	Hardness (g)	Cohesiveness	Adhesiveness (g/s)	Springiness	Gumminess (g)
*N* wheat starch	71.6 ± 1.6^a^	3,758±48^d^	7,034±34^d^	1,214±84^d^	3,576±38^a^	56.8 ± 0.8^a^	62.0 ± 1.4^a^	67.4 ± 1.3^a^	11.8 ± 0.8^a^	1,420 ± 34^a^	0.93 ± 0.05^a^	−234 ± 13^a^	1.18 ± 0.09^a^	1,321 ± 85^a^
AC wheat starch (25 kHz)	58.3 ± 1.4^c^	7,188±32^b^	8,851±31^b^	3,102±18^b^	1663±53^d^	48.2 ± 1.0^c^	53.4 ± 1.3^c^	59.4 ± 1.84^c^	6.4 ± 0.8^c^	634 ± 28^c^	0.87 ± 0.05^a^	−312 ± 38^c^	0.71 ± 0.04^c^	852 ± 34^c^
AC wheat starch (40 kHz)	64.4 ± 1.2^b^	5,846±65^c^	7,648±66^c^	2,492±16^c^	1802±67^b^	51.0 ± 0.8^b^	57.6 ± 1.2^b^	62.1 ± 1.4^b^	9.5 ± 0.4^b^	896 ± 48^b^	0.91 ± 0.03^a^	−281 ± 24^b^	0.95 ± 0.08^b^	936 ± 52^b^
AC wheat starch (25 + 40 kHz)	50.9 ± 1.2^d^	9,423±112^a^	11,174±131^a^	4,011±24^a^	1753±53^c^	44.9 ± 1.4^d^	50.8 ± 1.1^d^	54.6 ± 1.8^d^	5.8 ± 0.8^d^	574 ± 31^d^	0.86 ± 0.09^a^	−327 ± 30^d^	0.62 ± 0.07^d^	616 ± 43^d^

Note

Different letters in each column show significant statistical difference between the values (*p* < 0.05).

### 3.8 Thermal behavior

Table 1 summarizes the DSC gelatinization parameters including transition temperatures (To, Tp, and Tc) and enthalpy of gelatinization (ΔH gel) related to N and AC (under sonication at frequencies 25, 40, and 25 + 40 kHz) wheat starches. It was observed that these values were lower in AC at frequency 25 + 40 kHz compared with AC at frequency 25 and 40 kHz and N starches. Modification induced a major reduction from 56.8 ± 0.8°C for N wheat starch to 48.2 ± 1.04°C, 51.0 ± 0.8°C, and 44.9 ± 1.4 for AC wheat starches at frequencies 25, 40, and 25 + 40 kHz, respectively. Among the starches, N starch presented the highest ΔH_gel_ value (11.8 ± 0.8 J/g), followed by AC wheat starches at frequency 25 kHz (6.4 ± 0.8), 40 kHz (9.5 ± 0.4), and 25 + 40 kHz (5.8 ± 0.8). Singh et al. (2004), Babic et al. (2007), and Lee and Yoo (2009) also reported a statistically significant (*p* < 0.05) decrease in the gelatinization temperature of corn, potato, tapioca, and sweet potato starches following acetylation. The starch gelatinization is controlled by two factors: (a) amylopectin molecular structures such as molar mass, integrity and order of crystallites, polydispersity, and amylopectin chain length with a degree of polymerization (DP) ranging between 5 and 12, and (b) granule structures including granule size, amylose/amylopectin ratio, crystalline zone relationship, and the quality and amount of amorphous crystallites (Tester & Morrison, 1990). The enthalpy value attributed to the ordered double‐helix loss was more than the crystallinity loss. The difference in enthalpy value between the N and modified wheat starch showed that the acetylation of wheat starch with a high substitution degree generated disorganization as is shown by X‐ray, SEM, and RVA experiments. The reduction in the gelatinization parameters of acetylated starches was due to the insertion of acetyl and propyl oxide groups into the starch molecules, particularly into the amorphous areas. This further reduced the integrity of the amorphous and crystalline sections of starch granules and disrupted the inter‐ and intramolecular hydrogen bonds, destabilizing the granular structure (Babic et al., 2007). ΔH indicates an overall measure of crystallinity and loss in the molecular order. The esterifying agents reduce ΔH due to (a) the change in the amylopectin double helices, (b) diminished crystallinity, (c) increase in starch chain flexibility, and (d) reduction in gelatinization temperature (Halal et al., 2015). These findings are consistent with Halal et al. (2015); Bello‐Pérez et al. (2010); Mirmoghtadaie et al. (2009); Kaur, Singh, and Singh (2004).

### 3.9 The effects of modification on starch morphology

**Figure 5:**
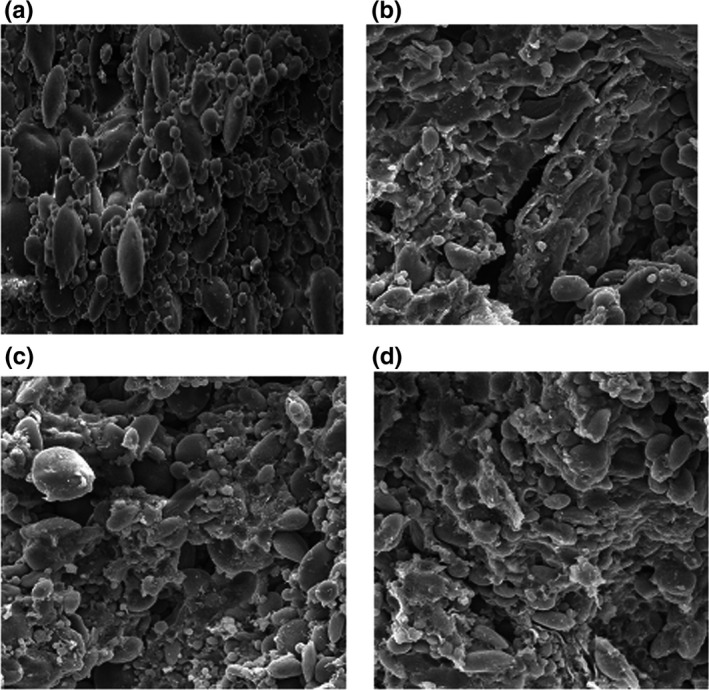
Scanning electron microscopy (*SEM*) of native (a) and acetylated wheat starch at frequencies 25 kHz (b), 40kHz (c), and 25 + 40 kHz (d)

The morphological patterns of N and modified starches are shown in Figure 5 where A and B granules of wheat starch can be clearly observed. In AC wheat starches at dual frequency (25 + 40 kHz), the surfaces of all large granules were more damaged after modification, compared with those of the small granules. Meanwhile, at frequency 25 and 40 kHz of wheat starch, small granules were more affected than large granules. Starch granules indicated a partial disintegration of granules with a slight gelatinization when esterification reactions were performed in the presence of catalyst (NaOH solution; Halal et al., 2015). According to Hirsch and Kokini (2002), the cross‐linked bonds formed by acetic anhydride mostly occur on the surface of the starch granules.

Modification by ultrasonication influenced the granule morphology through forming deep grooves in the central core zone, cracks on the starch granules, and blister‐like and doughnut‐like shapes, which is in line with the SEM morphology of Kaur et al. (2004). The major impact of acetylation on the surface of the granules is due to the reaction of acetic anhydride with starch granules under ultrasonic processes. Ultrasonic‐induced cracks, pores, and fractures on granular surfaces facilitate the penetration of chemical reagents into structure granules. According to the results of Bello‐Pérez et al. (2010), esterification of starch generated higher granule fusion, which is attributed to the increase in hydrogen bonding due to the insertion of hydrophilic groups into the starch molecules. Accordingly, coalescing starch molecules induce the fusion of starch granules (Bello‐Pérez et al., 2010), which is in line with the SEM morphology in the present study (Figure 5). The SEM results are also consistent with Van Hung and Morita (2005a, b).

### 3.10 Gel texture

Table 1 demonstrates the texture profiles of N and AC wheat starches under various frequency ultrasounds. The order of the hardness of AC wheat starch was as follows: 25 + 40 kHz < 25 kHz < 40 kHz. Acetylation was able to reduce the hardness of the gels due to (a) the introduction of large and hydrophilic acetyl groups which aided ultrasonication via increasing the space between the starch chain molecules and reducing retrogradation, (b) slight depolymerization of the starch molecules by the acetic anhydride under sonication (although more amylose molecules are reassociated, the depolymerization can weaken the gel), and (c) acetyl group preventing the formation of amylose gel network and the reassociation of amylose and amylopectin molecules (reducing retrogradation). There were no significant differences (*p* < 0.05) between N and AC wheat starches as far as cohesiveness is concerned; however, their gumminess was reduced. Adhesiveness is the attraction between the gel and an external surface. Adhesiveness of N wheat starch gel was significantly (*p* < 0.05) higher than AC wheat starch gels, which is in line with Liu et al. (1999), Choi and Kerr (2003), Babic et al. (2007), and Colussi et al. (2015).

### 3.11 Freeze–thaw stability

**Figure 6:**
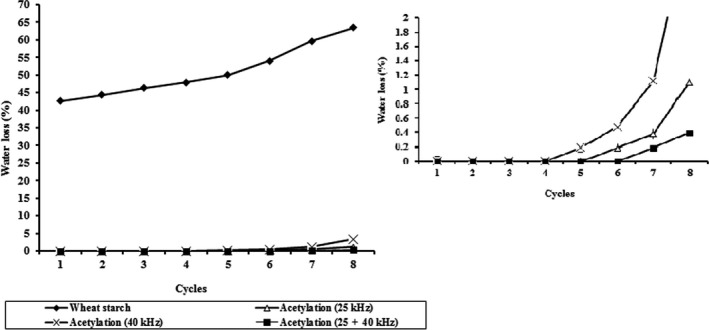
Freeze and thaw stability profile in N and AC wheat starch

The purpose of the present research was to stabilize the starch gels from syneresis during freezing and thawing. The tendency to retrogradation of gels prepared from N and AC wheat starches was calculated by determining the syneresis (percentage of water loss) during storage at 4°C (Figure 6). During the freezing cycles, the crystal size was enlarged with the reduction in their number. In the thawing stage, the ice crystals were converted to water masses easily removed from the polymer network (syneresis) (Smith & Schwartzberg, 1985). Furthermore, water leakage led to the formation of sponge texture in starch. N starch gels started to retrograde following 24 hr, indicated by the increase in the percentage of water from 42.64% to 63.47% during storage (8 cycles). Acetylated starch gels prevented the separation of water from starch noodle even after five freeze–thaw treatments, showing a better freeze–thaw stability due to high DS. The percentage of water separated from AC starch gels at frequencies 25 kHz, 40 kHz, and 25 + 40 kHz reached 1.10%, 3.36, and 0.39%, respectively, indicating the lower increment in syneresis compared with the N wheat starch gel, as illustrated in Figure 6. The highest resistance to freeze–thaw cycle(s) was observed in AC wheat starch at frequency 25 + 40 kHz (lowest % separated water), while the lowest belonged to N wheat starch (highest % separated water). Syneresis in freeze‐thawed gels is due to the reassociation of amylose molecules together and with amylopectin chain in starch granules at reduced temperatures which accelerates the exclusion of water from the gel structure. Amylose association takes place at the initial step of storage, while amylopectin association occurs in the following stages. Retrogradation may occur when a gelatinized starch is cooled. The increase in the amount of separated water indicates the lack of freeze–thaw stability. After acetylation, water molecules were absorbed by functional groups and were not able to freeze, thereby reducing syneresis (Han et al., 2012). On the other hand, the introduction of large groups of acetyl into the starch chains reduced the inner and outer bonds. Moreover, the acetyl group is smaller in size, which is effective on water absorption and stability against water exclusion. By inserting the acetyl group, the hydrophilic nature of these reagents resulted in the electrostatic repulsion between the chains, preventing the amylose–amylose and amylose–amylopectin interactions (Kaur et al., 2004; Lawal, 2010). When the amylose molecules carrying acetyl group are mingled with amylopectin, the acetyl group sterically prevents the aggregation of amylopectin, entailing lower retrogradation and reduction in the percentage of water separated during freeze–thaw cycles (Kaur et al., 2004; Lawal, 2010).

## 4 CONCLUSIONS

In order to increase the DS and consumption of minimum reagents, a reaction mixture was carried out under ultrasonic processes. The molar substitution optimizing AC wheat starch was 1.22%, 1.37%, and 1.02% for 25 kHz, 25 + 40 kHz, and 40 kHz, consuming 5% of acetic anhydride. High DS is due to the ultrasonic conditions at dual frequency (25 + 40 kHz) which form fractures, pores, and cracks on the surface of granules, facilitating the penetration of reagents into granule starch. Acetylation ameliorates the solubility, water absorption, resistance to freeze and thaw cycle, and viscosity peak, and reduces the gelatinization temperature.

## CONFLICT OF INTEREST

The authors declare that they do not have any conflict of interest.

## ETHICS STATEMENT

Human and animal testing is unnecessary in this study.
